# The Influence of Eating Habits on Type 2 Diabetes in Saudi Arabia: A Systematic Review

**DOI:** 10.7759/cureus.42638

**Published:** 2023-07-29

**Authors:** Osama M Almutairi, Tameem A Alhomaid, Abdulaziz M Alshuaibi, Rakan M Ahmad Alahmad, Norah H Al Mardhamah, Turki Alamri

**Affiliations:** 1 Family and Community Medicine Department, Faculty of Medicine in Rabigh, King Abdulaziz University, Jeddah, SAU; 2 Family Medicine, Qassim Health Cluster, Buraydah, SAU; 3 Family Medicine, King Fahad Armed Forces Hospital, Jeddah, SAU; 4 Family Medicine, Diabetes Center, King Khalid Hospital, Najran, SAU

**Keywords:** diabetes, factors associated with diabetes, diabetes mellitus, dietary behavior, diet, eating habits

## Abstract

Several dietary factors are associated with an increased risk of diabetes in Saudi Arabia. The increasing consumption of processed and sugary foods, including fast food and sugary beverages, in recent decades along with the rising prevalence of diabetes indicate the necessity of exploring the influence of eating habits on diabetes in Saudi Arabia. That is why the association between eating habits and diabetes in Saudi Arabia has become a topic of increasing interest. Therefore, this systematic literature review aimed to explore the influence of eating habits on the prevalence of diabetes in Saudi Arabia by providing a comprehensive synthesis of existing evidence from studies conducted on this topic in Saudi Arabia. A systematic search was conducted using predefined search terms in electronic databases, including PubMed, Embase, Medline, Google Scholar, and Scopus. Studies investigating the relationship between eating habits and diabetes prevalence among the Saudi Arabian population were included. Data extraction was performed, and the quality of included studies was assessed using appropriate tools. The findings were synthesized and discussed. Understanding the association between eating habits and diabetes in Saudi Arabia is crucial for developing effective preventive and management strategies for diabetes and other non-communicable diseases and promoting healthier eating habits in Saudi Arabia.

## Introduction and background

Diabetes mellitus is a significant global health issue affecting 422 million people worldwide by 2022 and causing 1.5 million deaths each year [1]. The most common type is type 2 DM, which is associated with lifestyle changes that lead to insulin resistance. Type 2 DM has been increasing steadily over the decades and is expected to affect more than 552 million people by 2030 [2]. DM is a metabolic disease characterized by high blood sugar levels due to problems with insulin secretion, insulin action, or both, and disrupted metabolism of carbohydrates, fats, and proteins [2,3]. This can lead to several health problems, including heart disease, stroke, blindness, kidney failure, and immune suppression.

Saudi Arabia has experienced a rapid increase in diabetes rates in recent decades, with the disease now considered a major public health concern in the country [4]. In 2022, it was estimated that 17.1% of adults in Saudi Arabia had diabetes [5], significantly increasing from the 2.1%-9% prevalence in the 1980s [6]. Though several factors contributed to this trend, including changes in lifestyle, urbanization, and shifts in dietary patterns, in Saudi Arabia, eating habits are among the most important factors influencing the risk of developing diabetes [7]. A number of studies have shown that people who eat unhealthy diets are more likely to develop diabetes. These diets are rich in processed foods, sugary drinks, and unhealthy fats [8-10]. These diets lack fruits, vegetables, and whole grains. In contrast, people who eat healthy diets high in fruits, vegetables, and whole grains are less likely to develop diabetes [11,12].

One major contributor to diabetes prevalence is consuming processed and sugary foods [13]. Saudi Arabia has changed as the country has undergone rapid expansion and modernization [14]. This has led to increased consumption of processed foods, which are high in calories, unhealthy fats, and sugars. This has led to an increase in type 2 DM, obesity, and cardiovascular problems [15,16].

This systematic review examined the evidence on the influence of eating habits on diabetes prevalence in Saudi Arabia, and its findings would help inform public health policies and interventions aimed at reducing the prevalence of diabetes in Saudi Arabia.

## Review

Methods

Study Design

This systematic review was conducted adhering to the systematic approach outlined by the Preferred Reporting Items for Systematic Reviews and Meta-Analyses (PRISMA) guidelines [17]. To identify relevant articles, we explored studies answering the following question: what is the influence of dietary, eating, or food habits on the prevalence of diabetes in Saudi Arabia?

Search Strategy

The following electronic databases were searched using specific search terms: PubMed, Embase, Medline, Google Scholar, and Scopus. These terms encompassed concepts related to eating habits, diabetes, and Saudi Arabia. We combined these terms using Boolean operators ("AND," "OR") to create search queries (e.g., “eating habits" OR "dietary patterns" AND "diabetes" OR "diabetes mellitus" AND "Saudi Arabia" OR "KSA", “food consumption" OR "nutritional habits" AND "type 2 diabetes" OR "hyperglycemia" AND "Saudi Arabia" OR "Middle East", "food choices" OR "dietary intake" AND "diabetes prevalence" OR "insulin resistance" AND "Saudi Arabia" OR "Gulf countries").

We performed manual searches of reference lists and gray literature to identify any additional studies of interest. We only included studies conducted and published within the last 10 years to account for the most recent advances in medical discoveries and technology.

Studies meeting the following criteria were included:

a) Population: Studies conducted among the Saudi Arabian population involving adults (>18 years old people).

b) Exposure: Investigation of eating habits or dietary patterns and diabetes.

c) Outcome: Assessment of diabetes prevalence or incidence and type 2 diabetes prevalence.

d) Study design: Observational studies (cohort, cross-sectional, case-control), intervention studies, systematic reviews, and meta-analysis articles.

e) Language: Studies should have been published in English, which is the academic and scientific language used in Saudi Arabia.

We excluded theses, editorials, commentary, opinion articles, narrative reviews, and other articles published in non-peer-review journals.

Study Selection

**Figure 1:**
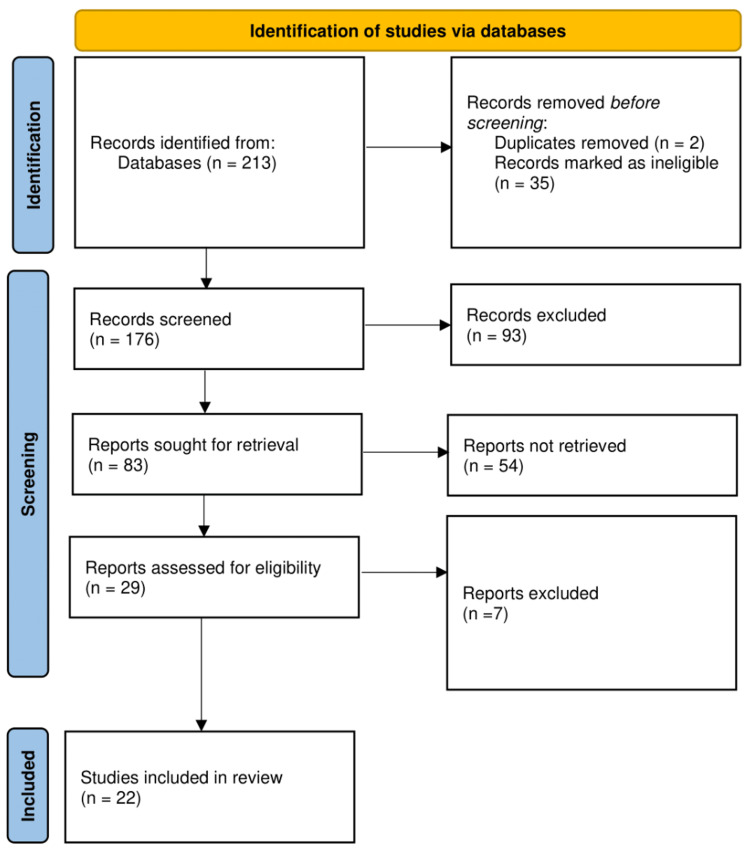
Flow diagram of the selection of studies included in our systematic review

Two independent reviewers screened the searched study titles and abstracts to determine their eligibility for inclusion. We considered all studies exploring eating habits or dietary patterns concerning diabetes prevalence or incidence rates in Saudi Arabia among adults. Figure 1 shows the selection of the studies included in the systematic review. Full-text articles were retrieved for potentially relevant studies, and any disagreements between reviewers were resolved through discussion or, if necessary, by involving a third reviewer.

Data Extraction and Quality Assessment

Two reviewers independently extracted data using a predefined extraction form, recording the following characteristics: authors, publication year, study design, and summary of findings. The quality of included studies was assessed by evaluating the sample representativeness, measurement validity, and potential biases using appropriate tools based on the study design. For cohort studies, we used the Newcastle-Ottawa Scale (NOS) [18], while the Cochrane Collaboration's Risk-of-Bias tool (CCRB) was used for intervention studies [19]. The NOS, with a scale of 0 to 9, is used to assess the quality of cohort studies. Except for comparability, which allowed for two points, each NOS item could only receive one point, with a minimum score of zero [18]. The Agency for Healthcare Research and Quality Scale (AHRQ), with scores ranging from 0 to 11, was used to assess the quality of cross-sectional research, with higher ratings showing better research methodology [20]. The CCRB consists of specific domains that assess random sequence generation, allocation concealment, blinding of participants and personnel, blinding of outcome assessment, incomplete outcome data, selective reporting, and any other potential sources of bias specific to the study design [19].

Data Synthesis

We synthesized the findings by summarizing and categorizing them based on key themes identified in the included studies and presenting them in tables. By considering the strength of evidence, consistency of findings, and limitations of the included studies, we discussed the influence of eating habits on diabetes prevalence in Saudi Arabia.

Results

The first search yielded 213 results. A total of 219 titles and abstracts were checked after duplicates were removed, and 109 studies were assessed for full-text screening. In total, 35 articles were deemed eligible and subjected to a thorough review. Following a thorough analysis, only 22 studies were included in our systematic review (Table 1). Of the 22 studies included, most (17) were cross-sectional, three were cohort studies, one was a systematic review, another was a meta-analysis, and one was a case-control study.

**Table 1:** Characteristics of the included studies DM, diabetes mellitus; HbA1c, hemoglobin A1c; HDL, high-density lipoprotein; OR, odd ratio; BMI, body mass index

Authors	Year of publication	Title	Study design	Summary of findings
Aljahdali and Bawazeer [21]	2022	Dietary patterns among Saudis with type 2 diabetes mellitus in Riyadh: a cross-sectional study	A cross-sectional study	Identified five dietary patterns were: "vegetables and olive oil," "refined grains and sweets," "dairy products and legumes," "dates and beverages," and "fruit." These patterns were associated with various sociodemographic, lifestyle factors, and eating behaviors, including age, gender, smoking, physical activity, number of snacks consumed, and frequency of meals in fast food locations and restaurants.
Alrasheedi [22]	2018	Evaluation of dietary habits effect among Saudi patients with type II diabetes mellitus	Cross-sectional study	A half of the participants had an excellent level of diet knowledge and skills. The majority (71.0%) had no eating issues, and 47.5% had no diet barriers.
Alramadan et al. [23]	2018	Glycaemic control for people with type 2 diabetes in Saudi Arabia - an urgent need for a review of the management plan	Cross-sectional study	Approximately 75% of participants had poor glycemic control (≥7%). Factors such as age ≤ 60 years, longer DM duration, remote location, low income, unhealthy diet, lack of knowledge, and medication issues contributed to inadequate control.
Alassaf et al. [11]	2021	Dietary habits of Saudi Medical Students at King Saud Bin Abdulaziz University for Health Sciences in Riyadh, Saudi Arabia	Cross-sectional study	The male group consumed fast foods at a higher rate (95.2%) than the female group (80.7%). Fast foods were predominantly consumed around dinnertime by both genders. The male and high-income groups consumed more fried meals and pastries than the female and low-income groups (64.1% and 66.7% vs. 50% and 45.7%, respectively, p<0.05).
Sabur et al. [24]	2022	Determinants of healthy food consumption and the effect of Saudi food-related policies on the adult Saudi population, a national descriptive assessment 2019	Cross-sectional study	A significant percentage (43.2%) did not meet dietary recommendations in any food group, while only 1.53% followed recommendations for all groups. Nearly half (47.8%) did not engage in physical activity. Food preferences varied, with 34.7% preferring healthy food, 18.8% preferring unhealthy food, and 46.5% having mixed preferences.
ALFaris et al. [25]	2015	Trends of fast food consumption among adolescent and young adult Saudi girls living in Riyadh	Cross-sectional study	Among participants, 95.4% consumed fast food from restaurants, with 79.1% eating it weekly. Girls preferred burgers and soft drinks. Larger fast-food portions were associated with higher waist and hip circumference. Taste and convenience drove fast food consumption, while 62.2% of girls expressed concerns about hygiene and safety. International restaurants were favored over local ones (70.9% vs. 29.1%).
Sami et al. [8]	2020	Dietary attitude of adults with type 2 diabetes mellitus in the Kingdom of Saudi Arabia: a cross-sectional study	Cross-sectional study	Patients exhibited an overall inappropriate dietary attitude, including food selection, healthy choices, food restraint, health impact, and food categorization. They also had a poor attitude toward consuming red meat, rice, soup and sauces, dairy products, and junk food.
Sami et al. [26]	2021	Food consumption and lifestyle habits among university students in Saudi Arabia	Cross-sectional study	Approximately 46% of males and 55% of females consumed fast food weekly, while 30% of males and 24% of females had 1-2 fast food meals monthly. Skipping meals was common, especially breakfast. Female students had a higher meal-skipping rate (72%). Many male students (72%) reported low vegetable and fruit intake. Convenience was the primary reason for fast food consumption among males (31%), while females cited choice (32%). Female students exhibited higher nutrition knowledge scores than males.
Al-Mountashiri et al. [12]	2017	Dietary habits, physical activity and diabetes perception among patients with type 2 diabetes mellitus in Tabuk City, Saudi Arabia	Case-control study	BMI (p<0.001), fast food intake (p<0.001), fruit consumption (p=0.004), and breakfast skipping (p<0.001) were positively associated with DM patients. Poor DM control was also associated with sweet food consumption (p=0.046).
Alwadeai and Alhammad [5]	2023	Prevalence of type 2 diabetes mellitus and related factors among the general adult population in Saudi Arabia between 2016–2022: a systematic review and meta-analysis of the cross-sectional studies	Meta-analysis	This meta-analysis of studies from 2016 to 2022 showed that the prevalence of type 2 DM was 28%, and the risks of type 2 DM in people over 40 years of age were nearly twice as high (OR = 1.74, 95% CI = 1.34-2.27) than in people under 40.
Li et al. [27]	2022	Dietary patterns derived from reduced rank regression are associated with the 5-year occurrence of metabolic syndrome: Aichi Workers’ Cohort Study	Cohort study	Healthy dietary pattern was negatively associated with metabolic syndrome (p=0.009) and hypertension (p= 0.002). In contrast, an unhealthy diet was linked with metabolic syndrome and its components (p<0.05).
Ilamathi et al. [28]	2023	Association between type of diet, extent of sweet consumption and status of physical activity with presence of type II diabetes mellitus	Cross-sectional analytical study	Participants who followed a vegetarian diet with less sugar consumption and greater physical activity were shown to have improved DM management.
Alali et al. [29]	2023	Dietary assessment of type 2 diabetic patients using healthful plant-based diet score in the Eastern Province of Saudi Arabia	Prospective cohort	A plant-based diet was associated with reduced triglyceride levels (-3.78%) and potentially increased HDL levels (1.87%) in Saudi Arabian type 2 DM patients. The prevalence of comorbidities was 11.3% for coronary artery disease, 6.2% for stroke, 3.3% for peripheral artery disease, and 8.4% for chronic kidney disease.
Amin et al. [30]	2014	Profile of non-communicable disease risk factors among employees at a Saudi University	Cross-sectional study	The prevalence of DM was 21.5% among employees of King Faisal University in Al Hassa, Saudi Arabia. Raised cholesterol was identified in 36.6% of the participants, low HDL in 36.8%, and raised triglycerides in 36.1%. Daily current smokers made up 22.7% of the questioned employees, 73% were physically sedentary, 64% were overweight or obese, and 22.1% had hypertension.
Alotaibi et al. [31]	2020	Incidence and prevalence rates of diabetes mellitus in Saudi Arabia: an overview	Systematic review	The study found that the prevalence of DM was 17.2% in Saudi Arabia. The study also found that eating habits were associated with DM prevalence, with a higher intake of processed foods and a lower intake of whole grains associated with an increased risk of DM.
Al Mansour [32]	2019	The prevalence and risk factors of type 2 diabetes mellitus (DMT2) in a semi-urban Saudi population	Cross-sectional study	The prevalence of type 2 DM among the semi-urban population of Saudi Arabia is high (34.6%). The disease is more prevalent among elderly respondents and is associated with obesity, high triglycerides, low HDL, and high total cholesterol.
Al-Mssallem et al. [33]	2020	Dietary pattern of patients with type 2 diabetes mellitus including date consumption	Cross-sectional study	This study showed a link between high date fruit consumption and reduced HbA1c and fasting blood glucose levels in people with type 2 DM.
Al-Mssallem et al. [34]	2020	Dietary carbohydrate intake in patients with type 2 diabetes mellitus and diabetes control: a cross-sectional study	Cross-sectional study	The findings showed that dietary carbohydrate intake exceeded the required daily requirement, with white rice (Basmati rice) being the primary contributor. However, dietary non-starch polysaccharide intake was lower than recommended, negatively associated with HbA1c levels.
Mirghani and Saleh [35]	2022	Diabetes risk among medical students in Tabuk City, Saudi Arabia	Cross-sectional study	Obesity and overweight were observed in 21.3-26.6% of students, 45.6% had central adiposity, and more than half did not exercise daily; 60.4% did not consume fruits and vegetables daily, and 16% had a high or moderate risk of DM
Al-Maiman et al. [36]	2016	Impact of fasting during Ramadan on daily habits, diet and body weight of individuals with diabetes: a sample of Saudi Arabia	Cohort study	Ramadan fasting had positive effects on weight for type 2 DM patients in Saudi Arabia. They generally did not engage in physical activity and increased calorie, carbohydrate, and fat intake but their anthropometric measures decreased.
Hanbazaza et al. [37]	2022	Relationship between food insecurity and diabetes among patients in Saudi Arabia	Cross-sectional study	Food insecurity was prevalent in 26.2% of participants, particularly among non-Saudis, unemployed individuals, and those with low incomes. It was associated with irregular blood glucose monitoring, cost-related nonadherence to medication, and hypoglycemia-related problems. Unhealthy food consumption mediated the relationship between food insecurity and hypoglycemia and its consequences.
Jambi et al. [38]	2020	The association between dietary habits and other lifestyle indicators and dysglycemia in Saudi adults free of previous diagnosis of diabetes	Cross-sectional study	In men, consuming 5 or more portions of red meat and Turkish coffee per week was associated with decreased odds of dysglycemia. In women, consuming fresh juice 1-4 times per week or 5 or more times per week was associated with decreased odds of dysglycemia. However, consuming a hibiscus drink 5 or more times per week was associated with increased odds of dysglycemia. Other lifestyle factors did not show significant associations with dysglycemia.

Prevalence of type 2 diabetes

The prevalence of type 2 DM was reported to be 27.6-34.6% [5,14,32]. A meta-analysis of studies from 2016 to 2022 showed that the prevalence of type 3 DM was 28%, and the risk among people over 40 years of age was nearly twice as high (OR = 1.74, 95% CI = 1.34-2.27) than in people under 40 years of age [5], which is higher than the prevalence of 21.5% reported among employees of King Faisal University in Al Hassa [14]. However, the semi-urban population of Saudi Arabia was found to have the highest prevalence of type 2 DM (34.6%) [32]. It was found that DM was more prevalent among elderly respondents and is associated with obesity, high triglycerides, low high-density lipoprotein (HDL), and high total cholesterol [14,32].

Eating habits and their association with diabetes

Nine articles reported on eating habits and dietary patterns. These include eight cross-sectional studies [8,11,21-26] and one case-control study [12]. One article reported that 43.2% of adults in Saudi Arabia did not meet dietary recommendations in any food group, and 18.8% preferred unhealthy food for convenience or ease of preparation and speedy delivery [24]. Male students at King Saud bin Abdulaziz University for Health Sciences in Riyadh consumed fast foods at a higher rate (95.2%) than women (80.7%), and those with high income were more likely to consume unhealthy diets made of fried meals than those in the low-income group (p<0.05) [11]. Studying adolescent and young adult Saudi girls living in Riyadh, it was found that 95.4% consumed fast food from restaurants and preferred burgers and soft drinks, also influenced by taste and convenience. However, 62.2% of girls expressed concerns about hygiene and safety [25]. Another study by Sami et al. [26] also reported convenience as a driver of unhealthy food consumption, and they found that 46% of male and 55% of female students consumed fast food weekly and that 72% of students reported low vegetable and fruit intake. Another study evaluating adults with type 2 DM found poor dietary attitudes toward food selection, healthy choices, food restraint, health impact, and food categorization [8]. On the other hand, it was found that the sociodemographics of patients with type 2 diabetes, such as eating behaviors, number of snacks consumed, and frequency of meals in fast food locations and restaurants, were associated with dietary categories/patterns, including vegetables and olive oil, refined grains and sweets, dairy products and legumes, dates and beverages, and fruit [21]. One study evaluating trends of fast-food consumption reported that international restaurants selling more fast food were favored over local ones (70.9% vs. 29.1%) [25].

The association between unhealthy diet and type 2 diabetes or glycemic control was reported in nine studies, including six cross-sectional studies [28,33-35,37,38], one prospective cohort [29], one case-control [12], and one systematic review [31]. The influence of diet on diabetes or glycemic control was identified, with an unhealthy diet contributing to a high risk of type 2 DM [21-23]. One study reported that three-quarters of patients with type 2 DM had poor glycemic control, influenced mostly by an unhealthy diet [23]. A case-control study on patients with type 2 DM in Tabuk City, Saudi Arabia, showed that fast food intake (p<0.001), fruit consumption (p=0.004), and breakfast skipping (p<0.001) were positively associated with diabetes, while sweet food consumption was negatively associated with diabetes control (p=0.046) [12]. One cohort study reported that a healthy diet was negatively associated with metabolic syndrome (p=0.009) and hypertension (p= 0.002), while an unhealthy diet was associated with all of its components (insulin resistance, high blood triglycerides, low levels of HDL cholesterol, obesity, and high blood pressure) (p<0.05), increasing risks for type 2 DM [27]. Two articles (one cohort and one cross-sectional) reported a positive association between a plant-based diet and good diabetic management [28], reduced triglyceride levels, and increased HDL levels [29]. Two cross-sectional studies found that high date fruit and non-starch polysaccharide consumption reduced HB1c levels, indicating better long-term glycemic control [33,34]. One systematic review found that eating habits were associated with diabetes prevalence, with a higher intake of processed foods and a lower intake of whole grains being associated with an increased risk of diabetes [31]. Fresh juice, fruits, and vegetables were found to reduce the risk of diabetes and deglycation by three studies [35,37,38]. One study evaluated the impact of fasting during Ramadan on the diet of patients with diabetes and found that Ramadan fasting had positive effects on weight for type 2 DM patients in Saudi Arabia despite increased calorie, carbohydrate, and fat intake [36]. Another study found that unemployment, non-Saudi nationality, and low income were associated with unhealthy diets, food insecurity, and poor glycemic control among patients with type 2 DM [37]. A study evaluating diet knowledge among diabetic patients found that 50% had excellent diet knowledge and skills, most (71.0%) had no eating issues, and 47.5% had no diet barriers. Another study reported the lack of knowledge as a factor for poor glucose control [23].

Discussion

In Saudi Arabia, diabetes has become a significant health issue [4]. While the role of eating habits in diabetes development and management is well-established, the specific influence of these habits on diabetes prevalence in Saudi Arabia remains understudied [12,21]. Addressing this knowledge gap is crucial for designing effective preventive and management strategies. Our systematic literature review aims to bridge this gap by examining the available evidence regarding the impact of eating habits on diabetes in Saudi Arabia.

This review showed that the prevalence of DM in Saudi Arabia is high, affecting a third of the adult population in general. This places Saudi Arabia among the countries with the highest diabetes prevalence in the world [31]. This prevalence is higher than the 23.7% reported in Saudi Arabia a decade ago, placing Saudi Arabia among the top 10 countries with the highest diabetes burden [39]. Studies have reported that the urban population was the most affected [39], which our review confirmed is still the case.

Several factors contribute to this increasing trend, including changes in lifestyle, urbanization, and shifts in dietary patterns [32,38]. Eating habits that play a significant role in the prevalence of diabetes in Saudi Arabia, as in many other countries, have changed as Saudi Arabia underwent socio-economic changes [7]. This review showed that most Saudis did not meet dietary recommendations, and fast food and processed diet consumption was popular, especially among young Saudis and students, mainly due to convenience and easy access. This aligns with previous research showing that one of the key factors influencing diabetes prevalence in Saudi Arabia was the high consumption of processed and sugary foods due to their availability and affordability [15,16]. Mousa and Ahmed [40] reported that 89.2% of the Saudi community population ate fast food. However, 88.4% encouraged their friends to buy a healthy diet, and the motivation was its taste and speed. However, they reported a desire among the community to stop consuming unhealthy food, indicating their awareness of the dangers associated [40].

Once rich in whole grains, fruits, and vegetables, traditional Saudi diets have been replaced by diets high in refined carbohydrates, saturated fats, and added sugars [4,41]. This shift can be attributed to the easy availability and affordability of processed foods, fast food chains, and sugary beverages, as shown by our review. Traditional Saudi Arabian diets were relatively healthy, consisting of dates, whole grains, legumes, and lean meats. However, the influence of Western diets has led to a shift toward consuming more processed foods, resulting in increased risks of diabetes and obesity in Saudi Arabia [9,10]. A previous review describing the effect of a Western diet on metabolism showed that it negatively affects the body's antioxidant status, immunity, gut microbiota and mitochondrial fitness, cardiovascular system, and mental health, which are the opposite of the effects of a healthier diet [42]. Our review supported the evidence showing that date fruits reduced the risks of diabetes by reducing HbA1c levels and fasting blood glucose levels. Previous studies showed that the Mediterranean diet, characterized by a limited red and processed meat intake and a high intake of vegetables, fruits, nuts, seafood, and olive oil that are protective against diabetes [11,12], keeps becoming less popular in Saudi Arabia in favor of fatty food and processed diet [13]. This aligns with our review showing that the increase in unhealthy diet consumption with an increase in diabetes as well as higher popularity of international restaurants selling fast food than local restaurants selling traditional meals. This review showed that a healthy diet rich in vegetables, fruits, and whole grains, with fewer calories, carbohydrates, and less fat, was associated with fewer risks of diabetes, better glycemic control, fewer triglycerides, and LDL (low-density lipoprotein) cholesterol levels, known to contribute to diabetes and other health problems, such as cardiovascular diseases. This aligns with other previous studies [15,16]. Supporting our findings, a study conducted in Iran found an association between fried-food consumption and the risk of type 2 DM [43]. This review found that sweet food consumption was negatively associated with diabetes control, while a plant-based diet reduced triglyceride levels, increased HDL levels, and led to better diabetes management. This is also similar to previous research showing that a diet rich in plant-based components reduced risks of non-communicable diseases (NCDs) including diabetes, while food could increase the NCD risks [13].

Several factors could lead to the consumption of an unhealthy diet in addition to convenience and taste, as shown by this review. These might include socio-economic factors, such as low income and unfamiliarity with a new environment in Saudi Arabia, especially for expatriates. Our review supports this by showing more unhealthy diet consumption and poor diabetes control among non-Saudis and low-income populations. Previous studies also support our findings by showing a positive association between low income and unhealthy diets as well as food insecurity, hindering access to more expensive healthy diets compared to affordable junk food [44,45]. A study conducted on Asian, Middle-Eastern, and North African immigrants in Saudi Arabia showed they are more likely to engage in unhealthy diets, leading to compromised health [46]. Knowledge was also highlighted as another factor influencing dietary habits [4,47]. This review showed that high diet knowledge and skills were associated with healthy diet consumption and that female gender was associated with high diet knowledge. Another previous study found most participants showed an average level of dietary knowledge in general, as well as poor knowledge of carbohydrates, fat, and poor food choices [47]. Similarly, this review found inadequate diet knowledge and practices more common among men than women. Studies showed that poor knowledge resulted in high consumption of fast food and was associated with high rates of metabolic syndrome and obesity [48,49], consistent with our review’s findings. Factors such as gender and age have been identified as influencers of dietary patterns and risks of diabetes in addition to marital status, education level, and income [45]. Our review showed that male gender and old age were associated with obesity, high triglycerides, low HDL, and high total cholesterol, which are risks of diabetes. Similar findings were reported by a previous cohort study in Taiwan [50].

Promoting public awareness about the importance of healthy eating habits and their impact on diabetes risk is crucial. In addition, establishing and implementing policies that discourage the consumption of unhealthy foods and promote the availability and affordability of fresh fruits, vegetables, and whole grains is recommended, as it effectively reduces the burden of diabetes and other NCDs [42]. Healthcare providers should actively educate patients about healthy eating habits and offer guidance on dietary choices. Access to diabetes screening, early diagnosis, and appropriate management and treatment are essential components in reducing the burden of diabetes [51]. Combining efforts from government agencies, healthcare professionals, educators, the food industry, and communities can lead to effective strategies for combating diabetes.

There are certain limitations to consider in this systematic review. There was a lot of diversity between the studies in terms of risk factor definitions, study designs, and demographic characteristics, and there were no consistent criteria for evaluating variables among studies that might affect relevant factors discovered. This review did not explore other factors contributing to diabetes beyond diet and did not explore typical diet meals most likely to contribute to diabetes in Saudi Arabia. This was a systematic literature review, which is prone to selection bias and statistical heterogeneity. Further studies are also recommended to resolve these limitations.

## Conclusions

In conclusion, eating habits influence the prevalence of diabetes in Saudi Arabia, with unhealthy diets leading to high rates of diabetes and associated comorbidities. This review showed that unhealthy diet practice was high, and their increase was influenced by processed food taste and convenience. Male gender, old age, and low income are associated with unhealthy eating, poor glycemic control, and a high risk of diabetes. By addressing the influence of unhealthy eating habits on diabetes prevalence and implementing comprehensive strategies, diabetes can be reduced in Saudi Arabia, and the Saudi population's overall health can be improved.
